# Typhoid Pneumonia—Its Treatment, &c.

**Published:** 1879-03-20

**Authors:** Alban S. Payne

**Affiliations:** Virginia


### Southern Medical Record.

EDITORS:

T. S. POWELL, M.D. W. T. GOLDSMITH, M.D. R. C. WORD, M.D

*R. C. WORD, M.D., Managing Editor.*

☞ All Communications and Letters on Business connected with the RECORD must be addressed to the Managing Editor.

VOL. IX. ATLANTA, GA., MARCH 20, 1879. No. 3.

### ORIGINAL AND SELECTED ARTICLES.

### *TYPHOID PNEUMONIA.—ITS TREATMENT, &c.*

BY ALBAN S. PAYNE, M.D., OF VIRGINIA.

[Concluded.]

From what I have previously said, I shall not surprise you when I declare, in my opinion, acute pneumonia, as well as pneumonia typhoides, is more often an *irritation* than an inflammation of the parenchymatous structure of the lungs, and is caused by a *stasis*, an engorgement of blood. That *stasis* is a step toward inflammation I freely admit, and if not relieved may end in disorganization or gangrene of the lung. That many, very many cases, if seen early, and are treated promptly, boldly, *can* be *aborted.* I say this boldly, frankly, after upwards of thirty years' active practice in Piedmont, Virginia, and after a diligent study of her climate, her soil, her hydrography, her topography, her geology, and a critical study of her diseases. It is known to all that to the sudden climacteric alternations incident to this fine region of country, pneumonias are in the spring, fall and early winter, of frequent occurrence, sometimes taking on an endemic character. I am very glad to know that very able men (assisted by microscopical investigations) have taken in hand this important and interesting subject, and to-day it is a closely disputed point whether inflammation is the consequence of *plus* vitality or of *minus* vitality. Typhoid pneumonia is pneumonia with primary symptoms of a typhoid or asthenic type, and marked by rapid failing of vital powers. The crepitant rale is less frequent, less abundant. There is, however, such manifest prostration attending typhoid pneumonia, both mental and physical, from its inception, that acute pneumonia could hardly be confounded with it. The cough in typhoid pneumonia also may be said to be peculiar, being more of an effort to cough than "coughing". But the chief symptoms to rely on in distinguishing a case of pneumonia typhoides from the other affections of the lungs are the heavy, dull expression of the eyes, the oppressed intellect, muttering delirium, subdued muscular power and a feeble pulse. Bilious pneumonia is pneumonia, sometimes sthenic, sometimes asthenic, wearing the livery of malaria. Bilious pneumonia is regarded by some writers as nothing more than a special form of remittent or intermittent fever, in which the lungs are made to bear the burden of the disease. It is held by others to be a variety of pneumonia, occasioned by the ordinary causes of pneumonia, but owing its peculiar symptoms to its happening in those in whose systems the poison of malaria has been slumbering. Typhoid pneumonia could hardly be confounded with either pleuro-pneumonia or with broncho-pneumonia, unless the broncho-pneumonia was a complication in a case of typhoid fever. In this case, whilst the diagnosis might be somewhat difficult, the treatment would be the same in both diseases, bearing in mind that the case of broncho-pneumonia will recover more slowly than the case of typhoid pneumonia.

Rest to the mind and body is the *sine qua non* in almost every disease, particularly is this the case in typhoid diseases. In pneumonia, in every variety the patient should have absolute rest of mind and body; the lungs should be kept as still and quiet as an inflamed joint is ordered to be kept by all practitioners of medicine. Talking is as hurtful to the lungs, when in a diseased condition, as walking is to an inflamed knee joint. But in every variety of pneumonia you must have rest to the lungs and sleep. Now fortunately for us we can procure rest to the lungs and the other great desideratum in the treatment of pneumonia by the administration of the same remedy. If one remedy fails to meet these two great desideratums in typhoid pneumonia another must be substituted, for I tell you whilst talking must be controlled the patient must have sleep, sleep prolonged through the space of thirty-six or forty-eight hours, if found necessary. This sleep is often procured best by nerve-stimulants, ergo sedatives. The remedie sproducing nerve force are always soporific, given in full doses. I have seen as profound sleep produced by large doses of quinine as I ever did by the administration of opium. The engorgement of the pulmonary vessels is best gotten rid of by a large fly-blister over the branches of the great sympathetic nerve. This nerve transmits its own stimulation to the heart, forcing the heart to contract more powerfully upon its contents, and a freer current is thrown out through the vessels of the lungs (so to speak), which washes away the stagnating fluid, and your engorgement is relieved, and the quality of the blood itself is improved by this increased *vis a tergo* of the heart. Any pressure about the portal system should be removed, as it serves to throw "back water" upon the lungs and greatly embarrasses their proper functional action. These engorgements about the portal system are most successfully overcome by the administration of bilious purgatives, of which the admixture of submur. hydrarg. and compound ext. colycinth, with a small proportion of pulv. ipecac are the best.

However, the greatest desideratum in pneumonia is sleep, without which all treatment will prove of no avail, must be produced by large doses of quinine, or by a combination of quinine and pulvis doveri, or morphia or fluid extract of aconite. If there is an intolerance from idiosyncrasy to any one remedy, another having the same remedial virtues must be substituted in its place. If sulph. quinine is badly borne, give carbonate of ammonia in its place, and so on. We should never try to force a remedy on a peculiar idiosyncrasy in a time of serious sickness.

I have attempted to establish in this paper that venesection, the exhibition of antimonials, expectorants, etc., is not the best plan for any of the varieties of pneumonia; that in inflammations of the serous membranes venesection and the depleting remedies generally are always beneficial. In irritations and inflammation of mucous membranes, if ever beneficial at all, it is so as a mechanical remedy only. I again submit the question. If a large amount of poor blood cannot support the system, how can a smaller amount of the same furnish any better support? By the application of your lancet, you do not propose to improve the quality, but only to lessen the quantity. Again, whilst diseases affecting the "ganglionic" or "organic" nerves, are sthenic and will bear the depleting remedies with beneficial results, that where the ganglionic and cerebro-spinal system of nerves are both affected, the disease becomes asthenic, and will not stand, but will sink under the exhibition of depleting or reducing remedies.

Now as I think I have established satisfactorily to every candid reader that most pneumonias are not inflammations, but only a stasis of blood; that typhoid pneumonia is a disease in which the ganglionic system and the cerebro-spinal system of nerves are both implicated, and therefore belong to the type of diseases denominated asthenic, which we have seen will not be benefitted by the exhibition of reducing remedies. It follows, as a matter of course, we have simplified the treatment so much; that there is little more left for us to say than in typhoid pneumonia make your patient sleep, and sustain him by building-up remedies, so that he may *sleep* and enjoy nature's great restorer, healing sleep.

The remedial agents I have found the best in the treatment of typhoid pneumonia, are neither very costly nor numerous : quinine, submur. hydrargyrum, extract colycinth compound, ipecac, dovers powder, carbonate ammonia, green camphor, sulphate morphia, crab orchard salts, infusion eupatorium perfoliatum, fat and mush, tartar ointment, and a little alcohol in extreme cases being all I require. With these remedies I feel that I am not only able to cure every variety of pneumonia, but in many instances to "abort" the disease. In order that we may simplify this subject I will give a case or two from my note-book, representing the mild, average and most critical form of the disease under consideration.

I was called, January 3, 1875, to see George C——, a young, hearty, strong man aged nineteen years. Tongue not much furred, but trembling when attempted to put it out; pulse 110, very compressible; great and sudden muscular prostration; is unable to stand up; had a big chill four hours ago; short, hacking cough; is raising a glairy mucus streaked with blood; mind wandering, so much so, his father sent for me thinking he was going crazy; got very wet two days ago.

℞ Hot bricks to his feet, fat and mush poultice to the chest; as one poultice cools another hot to be applied every hour through the night. Sulphate quinine, grs. 5; submur. hydrarg., grs. 4; sulph. morphia, gr. ¼, hora somni; sulph. quinine, grs. 5, first thing in the morning, with teaspoonful crab orchard salts at 9 a. m.

January 4th, 12 m., bowels have been moved twice; cough fuller; breathing better; and expectoration easier and thicker; pulse 100; mind clearing up. ℞ Sulph. morphia, gr. ¼; sulph. quinine, grs. 5; ipecac, gr. ¼, hora somni; in the morning, sulph. quinine, grs, 5.

January 5th, 4 p. m. Slept well; tougue clean; mind clear; is up about the room; wants to eat. ℞ Sulph. quinine, grs. 5 at bed time and early in the morning; buttermilk half water ad libitum; glass warm toddy morning, noon and night. Was out on the street next day.

I was sent for to see Mrs. W——, December 25th, 1875. Is twenty four years of age; is the mother of one child; is English by birth; is of stout build and a brunette, but of a florid complexion; has had a long, severe attack of typhoid fever, since her arrival in America. She took a long walk about a week ago, to which she is unaccustomed. She has neither slept nor felt well since her fatiguing walk, but can't tell why; is not sensible of much pain; mind is clouded, wandering; pulse 145, very compressible; tongue, red and dry; it doubles up when she attempts to poke it out; whole posterior part of left lung in a state of hepatization; bowels running off; a short, dry, hacking cough. Had I

not known the fact that this lady had suffered from a long, severe attack of typhoid fever since she came to America, I believe I should have taken her case to be one of "typhoid fever," complicated with pneumonia. ℞ Sulph. quinine, grs. 5; pulvis doveri, grs. 5, to be taken every four hours, when not asleep; tartar ointment to be rubbed on the upper part of spinal column and between the shoulders; infusion eupatorium perfoliatum instead of water; room to be kept regularly warm and darkened; moist heated bricks to her feet, and bottles with warm water to be scattered around her in bed.

Dec. 26th. No change; prescription continued.

Dec. 27th. Pulse still 145; has slept a little; tartar begins to produce a crop of pustules. ℞ Continue the prescription and also rub ointment on anterior portion of the chest.

Dec. 28th. Has slept more; bowels stopped; pulse 105 and fuller; tartar ointment has produced a fine crop of pustules. Continue the prescription.

Dec. 29th. Has slept almost all night; begins to feel her weakness; for the first time asked for her child; has just found out she has been out of her mind; expectoration much improved; takes a little chicken soup, says it tastes good; looks on her chest and back as if she had just had the small pox.

Dec. 30th. Is much improved in every respect; is convalescing rapidly. ℞ Pulvis doveri, grs. x at bed time; sulph. quinine, grs. 5 ter in die sumendus; a glass of toddy whenever desired; chicken or squirrel soup ad libitum.

January, 2d. Is convalescent.

This lady after her recovery, still had a disposition to cough, and thinking she was threatened with consumption, she visited Colorado, remaining some six or eight months. In one month after her return she was again seized with typhoid pneumonia. I saw her early and soon "aborted" the disease, but after her recovery she again was annoyed with the cough. During my attendance in her last illness, I thought I discovered some taint of struma. I gave her five drops of tinct. iodine fort. in a syrup of sarsaparilla. It relieved the cough promptly, and she said had done more to restore her health than her trip to Colorado.

October 10th, 1855, I was called to see Whiting H——, about 45 years of age, a man with no surplus flesh, and naturally of a pale, cadaverous cast of countenance. I found on my arrival late in the day, that Dr. B——, of Clarke county, had been sent for also, and the doctor was on his horse just in the act of leaving when I arrived. I urged him to get down, but he persistently refused to do so, saying the patient was dying and he had the Shenandoah river to cross, and wished to cross it before it was dark. Left to my own resources, I surveyed the ground, determined to make the best fight I could with the disease. His wife informed me that his attack was brought on by over-exertion, fatigue, etc., etc. I must confess he had all the appearance of one in a fatal stage of collapse. I found him semi-comatose; pulse scarcely perceptible at the wrist; surface of the body, cold; countenance, pale, cadaverous, anxious; breathing, laborious, humid, decubitus, dorsal; unable to cough, much less to expectorate, although you could see where he had previously expectorated a rusty-brown colored sputa; his bed was found to contain a large quantity of thin serous-looking blood passed per anus. I locked up his bowels with an enema of water, starch and laudanum. I must have in this case full, free reaction, and I must have it quickly. He will not live until a blister can have its effect. What is to be done? Why, I said to myself, apply the actual cautery. I quickly rubbed his chest with alcohol and applied a lighted taper. The flash of the lurid light had hardly passed off before reaction came on; he became conscious; his breathing is *freer*, his pulse fuller and slower; his expectoration, easier, etc., etc. I gave him x grs. pulvis doveri with grs. 30 of quinine. In half an hour he was breathing much easier and sleeping sweetly. At 10 A.M. the next day he is almost convalescent; is quite cheerful and comfortable.

℞ Sulph. quinine, grs. 5; pulvis doveri, grs. 5, every four hours; buttermilk half water, or beef tea at will—continue for a few days. From this time convalescence rapidly supervened. No relapse. I heard lately he is enjoying good health in his new home in the state of Missouri.

